# Effects of thyroid hormone analogue and a leukotrienes pathway-blocker on renal ischemia/reperfusion injury in mice

**DOI:** 10.1186/1471-2369-12-70

**Published:** 2011-12-23

**Authors:** Najah R Hadi, Fadhil G Al-amran, Ayad A Hussein

**Affiliations:** 1 Department of pharmacology and therapeutics, Kufa medical college, Najaf kufa street, Najaf, Iraq; 2 Surgical department, Kufa medical college, Najaf kufa street, Najaf, Iraq

## Abstract

**Background:**

Acute renal failure (ARF) is an important clinical problem with a high mortality and morbidity. One of the primary causes of ARF is ischemia/reperfusion (I/R). Inflammatory process and oxidative stress are thought to be the major mechanisms causing I/R. MK-886 is a potent inhibitor of leukotrienes biosynthesis which may have anti-inflammatory and antioxidant effects through inhibition of polymorphonuclear leukocytes (PMNs) infiltration into renal tissues. 3, 5-diiodothyropropionic acid (DITPA) have evidences of improving effects on I/R in heart through modulation of cellular signaling in response to ischemic stress. The objective of present study was to assess the effects of MK-886 and DITPA on renal I/R injury.

**Methods:**

A total of 24 Adult males of Swiss albino mice were randomized to four groups: I/R group (n = 6), mice underwent 30 minute bilateral renal ischemia and 48 hr reperfusion. Sham group (n = 6), mice underwent same anesthetic and surgical procedures except for ischemia induction. MK-886-treated group: (n = 6), I/R + MK-886 (6 mg/kg) by intraperitoneal injection. DITPA-treated group: (n = 6), I/R + DITPA (3.75 mg/kg) by intraperitoneal injection.

After the end of reperfusion phase mice were sacrificed, blood samples were collected directly from the heart for determination of serum TNF-a, IL-6, urea and Creatinine. Both kidney were excised, the right one homogenized for oxidative stress parameters (MDA and GSH) measurements and the left kidney fixed in formalin for histological examination.

**Results:**

Serum TNF-α, IL-6, urea and Creatinine, kidney MDA levels and scores of histopathological changes were significantly (P < 0.05) elevated in I/R group as compared with that of sham group. Kidney GSH level was significantly (P < 0.05) decreased in I/R group as compared with that of sham group. MK-886 treated group has significantly (P < 0.05) lowered levels of all study parameters except for GSH level which was significantly (P < 0.05) higher as compared with that of I/R group. DITPA caused non-significant (P > 0.05) changes in levels of all study parameters as compared with that of I/R group.

**Conclusion:**

The results of the present study show that MK-886 significantly ameliorated kidney damage that resulted from I/R. For DITPA, as its administration might not be successful, administration using a different protocol may give different effects on I/R.

## Background

Acute kidney injury (AKI) is a common clinical syndrome characterized by rapid deterioration of renal function. AKI can be classified into three categories, namely, prerenal, intrinsic, and postrenal acute kidney injury. Progressive AKI leads to acute renal failure (ARF)[1]. ARF is an important clinical problem with a high mortality and morbidity. It affects 5% of hospitalized patients and has a mortality rate of approximately 50%[1]. One of the primary causes of ARF is I/R which is a drop in blood flow leading to inadequate supply of oxygen and nutrients to renal tissue which can be caused by, amongst others, surgery, organ transplantation and shock [1].

In injury due to ischemia, neutrophil stimulation with accompanying oxygen radical-mediated injury is the main event that leads to injury; under ischemic conditions, reduced oxygen supply leads to enhanced neutrophil adherence to tubular endothelial cells [2-4] due to increased surface expression of adhesion molecules on tubular endothelial cells [2,4,5]. This ultimately results, on reperfusion, in diapedesis of neutrophils and their oxidative burst, which results in oxygen radical production [6,7]. So in addition to the direct cytotoxic effects of hypoxia, renal I/R induces an inflammatory reaction within the renal parenchyma [8] by causing renal synthesis of pro-inflammatory cytokines such as Interleukin (IL)-1, IL-6, IL-18, and tumor necrosis factor (TNF)-α[9-12].

Many of the reactive oxygen species (ROS) produced by I/R activate the signaling mechanisms that culminate in TNF-α production [13]. TNF-α is a proinflammatory cytokine capable of up regulating its own expression, as well as the expression of other genes important in the inflammatory response [14]. TNF-α and I/R increase iNOS activity to synthesize nitric oxide [15,16]. Nitric oxide production (by iNOS) may play several roles in renal pathophysiology, including induction of tubular damage. Prevention or reduction of nitric oxide generation reduces nitric oxide-induced renal injury [17], and the increased generation of nitric oxide is capable of inducing intracellular oxidizing reaction and cell death [18].

I/R injury of the kidney is characterized by a series of events, including changes in vascular tone, enhanced vascular permeability to plasma proteins, structural alterations in renal tubule and accumulation of activated neutrophils [19]. The cysteinyl LT, LTC_4_, LTD_4_, and LTE_4_, affect the tonus of the arterioles and the permeability of postcapillary venules, thereby causing endothelial contraction and macromolecular leakage [20].

LTB_4 _is a mediator in the pathophysiology of the renal dysfunction caused by I/R of the kidney as well as the associated infiltration of the kidney with PMNs [21]. LTB_4 _activates PMNs, thus changing their shape and promoting their binding to endothelium by inducing the expression of cell-adhesion molecules. After PMN transmigration into ischemic renal tissue, PMNs release reactive oxygen species, proteases, elastase, myeloperoxidase (MPO), cytokines, and various other mediators [19], all of which exacerbate inflammation and contribute to tissue injury (positive feedback). For instance, ROS will react with the polyunsaturated membrane lipids [22], and lipid peroxidation, in turn, will enhance the tissue levels of free arachidonic acid [23].

A potent and selective inhibitor of 5-lipoxygenase activating protein (FLAP) is MK-886[24]which binds to FLAP with high affinity and prevents the activation of 5-lipoxygenase. MK886 is a highly selective compound with no effects on prostaglandin synthesis [25]. It does, however, inhibit translocation of 5-LOX [26].

Blocking leukotrienes was showen to be effective in improving I/R damage in liver, intestines [27] and also kidney (not by MK-886)[28] by inducing anti-inflammatory and antioxidant effects [27,28]. Therefore, in this study, we studied the effect of MK-886 on kidney injury and studied if MK-886 can exert anti-inflammatory and antioxidant properties through renal I/R injury.

Studies in animal and cell-based models give evidences that acute or chronic pretreatment with TH before I/R can protect from I/R injury [29]. The protective effect of acute T_3 _pretreatment was shown in an ex vivo canine heart preparation and in another study a model of rat heart in which cardiac work and cardiac efficiency were found to increase after no flow global I/R [30,31]. Similarly, long-term pretreatment with l-thyroxine for 2 weeks or T_3 _for 10 days was also shown to improve postischaemic recovery of function in isolated rat hearts subjected to zero-flow global I/R [32,33].

This protective effect may occur by attenuating the I/R-induced activation of the pro-apoptotic p38 Mitogen-activated protein kinases (MAPKs) by either negative regulation of Protein kinase C (PKCδ) or by decreased phosphorylation due to reduced ATP availability (due to reduced glycogen content which occurs during ischemia in hyperthyroid hearts) because stress kinases cannot be phosphorylated by upstream kinases without ATP [34].

3, 5-diiodothyropropionic acid (DITPA) is a synthetic TH-related compound with low metabolic activity which binds both nuclear TR isoforms with decreased affinity (Kd, 10^-7 ^M)[35]. In addition, DITPA binds integrin α Vβ3 through which it mediate angiogenesis [36]. Administration of DITPA after myocardial infarction in rabbits improved post-infarct recovery function [37]. Also In a rabbit post-infarction model, use of DITPA prevented abnormal sarcoplasmic reticulum Calsium transport and abnormal contractile function associated with myocardial infarction [38].

Based on these encouraging results about the effect of DITPA in experimental models of I/R in heart, in this study the effect of DITPA on I/R in kidney was studied.

## Methods

A total of 24 adult male of Swiss Albino mice (weighing 30-35 g, aged 12 weeks) were purchased from Animal Resource Center, the Institute of embryo research and treatment of infertility, Al-Nahrain University. All experiments were approved by the Animal Care and Research Committee of the University of Kufa, and this investigation conforms with the Guide for the Care and Use of Laboratory Animals (National Research Council). Animals were housed in the animal house of College of medicine/Al Kufa University in a temperature-controlled (24 ± 2°C) room with alternating 12-h light/12-h dark cycles and were allowed free access to water and diet until the start of experiments. After one week of acclimatization, the mice were randomized into four groups (6 mice in each group) as follow:

-Ischemia/reperfusion group: mice underwent ischemia for 30 minute then reperfusion for 48 hours.

-Sham group: mice underwent the same anesthetic and surgical procedures and received the same volumes of vehicles (for an identical period for ischemia and reperfusion) except for ischemia induction.

-MK-886-treated group: mice received MK-886 (Cyman chemical, USA) in a dose of 6 mg/kg by intraperitoneal injection, 30 min before the induction of ischemia, and the same dose was repeated just before reperfusion period. The drug prepared immediately before use as a homogenized solution in 2% ethanol [39] (not more than 0.1 ml was injected).

-DITPA-treated group: Mice received DITPA (Sigma chemical, USA) in a dose of 3.75 mg/kg by intraperitoneal injection, 30 min before the induction of ischemia, and the same dose was repeated just before reperfusion period. Solution of DITPA was prepared immediately before use by dissolving the powder in 0.1 N NaOH and diluting with 0.9% saline (pH 9)[40] (not more than 0.1 ml was injected).

### Induction of I/R

Animals were intraperitoneally anesthetized with 100 mg/kg ketamine and 10 mg/kg xylazine [41]. According to **Sharyo *et al *(2009)**[42], after anesthesia, an abdominal incision was made and the renal pedicles were dissected bilaterally. A vascular clamp (Biotechno, Germany) was placed on each renal pedicle for 30 min. After clamps were released, the incision was closed in two layers with 2-0 sutures and mice were returned back to their cages and left for 48 hr for reperfusion. Sham operations were conducted using the same procedure without placing a clamp on each renal pedicle. After the end of reperfusion phase mice were sacrificed using over dosing of anesthesia, blood samples were taken directly from the heart, both kidneys were excised, the right one homogenized then kept in deep freeze at - 80°C for oxidative stress measurement and the left kidney fixed in 10% neutral buffered formalin for histological examination.

### Inflammatory and Kidney Function Markers

At 48 hr after ischemia induction, (at the end of reperfusion period), from each mouse about 1.5 ml of blood was collected from the heart. The blood samples were allowed to clot at 37°C and centrifuged at 3000 rpm for 15 min; Sera were removed, and analyzed for determination of serum TNF-α, IL-6 (using ELISA kits of Immunotech, France), urea (using kit of BioMĕrieux^®^sa, France) and Creatinine (using kit of Spinreact, Spain).

**For IL-6**, Samples and calibrators were incubated in the microtiter plate coated with the first monoclonal antibody anti-IL-6, in presence of the second anti-IL-6 monoclonal antibody linked to acetylcholinesterase (ACE). After incubation, the wells were washed and the bound enzymatic activity is detected by addition of a chromogenic substrate. The intensity of the coloration is proportional to the IL-6 concentration in the sample or calibrator.

#### • Reagents provided

-Plate: 12 × 8 wells

-Calibrator: one vial contains lyophilized bovine serum albumin

-IL-6 ACE conjugate: one vial contains lyophilized bovine serum albumin

-Diluent 1: one 25 mL vial contains bovine serum albumin.

-Diluent 2: one vial contains lyophilized material of murine origin

-Wash solution (20×): one 50 mL vial

-Substrate: one vial

-Stop solution: Stop solution is a tacrine solution.

-Specificity: murine IL-6

- Dilution: firstly 1/10 then 1/3 for four times dilutions for standard (calibrator).

**For TNF-α**, Samples and calibrators are incubated in the microtiter plate coated with the first monoclonal antibody anti-TNF-α, in presence of the second anti-TNF-α monoclonal antibody linked to alkaline phosphatase. After incubation, the wells were washed and the bound enzymatic activity is detected by addition of a chromogenic substrate.

#### • Reagents provided

-Plate: 12 × 8 wells

-Calibrator: one vial contains lyophilized bovine serum albumin

-TNF-α conjugate: one vial contains lyophilized bovine serum albumin

-Diluent 1: one 25 mL vial contains bovine serum albumin.

-Diluent 2: one vial contains lyophilized material of murine origin.

-Wash solution (20×): one 50 mL vial

-Substrate buffer: one 30 mL vial of diethanolamine-HCl solution.

-Substrate: two tablets

-Stop solution: one 6 mL vial of NaOH 1 N solution.

-Specificity: murine TNF-α

-firstly 1/10 then 1/4 for three times dilutions for standard.

Oxidative Stress Measurement

The whole kidney tissues were homogenized with a high intensity ultrasonic liquid processor in 1:10 (w/v) 0.1 M potassium phosphate buffer (pH7.4). The 10% homogenates were centrifuged at 10, 000 rpm for 10 min at 4°C and supernatants were used for determination of GSH and MDA levels [43]. GSH kidney level (as an indices of antioxidant status) was measured using Quantichrom™Glutathione assay Kit (from BioAssay Systems, USA). MDA, the end product of lipid peroxidation, was analyzed according to the method of Buege and Aust in 1978[44] which based on the reaction of MDA with thiobarbituric acid (TBA) to form MDA-TBA complex, a red chromophore, which can be quantitated spectro-photometrically according to this method.

#### • Preparation of TBA reagent

-0.375 gm of TBA was added to 75 ml of DW.

- 15 gm of trichloroacetic acid (TCA) were added to DW.

- 2.1 ml of 11.9 N Hydrochloric acid (HCL) were added to DW.

- The solution completed up to 100 ml of DW.

#### • Procedure

-1 ml of kidney homogenate was added to 2 ml of TBA reagent and mixing.

-The mixture was heated by water bath at (100°C) for (15 min).

-Cooled and then centrifuged at 3000 rpm for (10 min).

- Light absorbance of clear supernatant was determined at 535 nm against blank using spectrophotomer.

#### • Calculation

The concentration of $\text{MDA}=\frac{absorbance\;at\;535\;nm}{\varepsilon }\times D$

*ε*: Extinction coefficient = 1.56 × 10^5 ^M^-1 ^Cm^-1^

D: Dilution factor

The results were expressed as nmol MDA/g tissue.

### Histopathological Evaluation

Renal sections were examined by light microscopy and scored according to a semiquantitative scale designed according to **Asaga *et al *(2008)**[45] to evaluate the severity of renal damage by a pathologist who was unaware of the treatment conditions. The slide section divided into 10 intersections. A score from 0 to 3 was given for each tubular profile involving each intersection as the following: 0 = normal histology; 1 = tubular cell swelling, brush border loss, nuclear condensation, with less than one-third of the tubular profile showing nuclear loss; 2 = same as for score 1, but greater than one-third and less than two-thirds of the tubular profile showing nuclear loss; and 3 = greater than two-thirds of the tubular profile showing nuclear loss. Then the total score for each kidney (section) was calculated by the summation of the all 10 scores for the all 10 intersections with a maximum Score of 30. According to the total severity score, the kidney injury was classified to normal (0), mild (1-10), moderate (11-20) and severe injury (21-30).

### Statistical analysis

Statistical analyses were performed using SPSS 12.0 for windows.lnc. Data were expressed as mean ± SEM. Analysis of Variance (ANOVA) was used for the multiple comparisons among all groups followed by post-hoc tests using LSD method. For the histopathological renal changes, the Mann-Whitney U was used to assess the statistical significance of difference between two groups in total severity score. In all tests, P < 0.05 was considered statistically significant.

## Results

### Effects on inflammatory parameters

**Figure 1:**
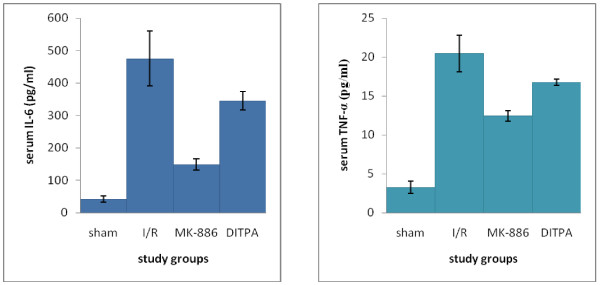
**Error bar charts show the difference in mean ± SEM values of serum IL-6 and TNF-α levels (pg/ml) in the four experimental groups at 48 hr after surgical operation (N = 6 in each group)**. * p < 0.05 when compared with sham group. ** p < 0.05 when compared with I/R group.

I/R caused significant (P < 0.05) elevation in serum TNF-α and IL-6 levels as compared with that of sham group. MK-886 significantly inhibited the elevated levels observed in I/R group. DITPA cause non-significant changes in serum TNF-α and IL-6 levels as compared with that of I/R group (see Figure 1).

### Effects on kidney function parameters

**Figure 2:**
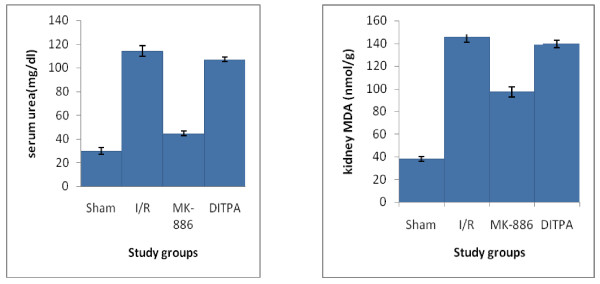
**Error bar charts show the difference in mean ± SEM values of serum urea and creatinine levels (pg/ml) in the four experimental groups at 48 hr after surgical operation (N = 6 in each group)**. * p < 0.05 when compared with sham group. ** p < 0.05 when compared with I/R group.

I/R caused significant (P < 0.05) elevation in serum urea and creatinine levels as compared with that of sham group. MK-886 significantly inhibited the elevated levels observed in I/R group. DITPA cause non-significant changes in serum urea and creatinine levels as compared with that of I/R group (See Figure 2).

### Effects on oxidative stress parameters

**Figure 3:**
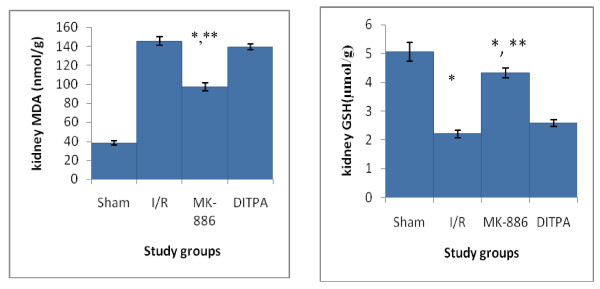
**Error bar charts show the difference in mean ± SEM values of kidney MDA (nmol/g_tissue_) and GSH (μmol/g_tissue_) in the four experimental groups at 48 hr after surgical operation (N = 6 in each group)**. * p < 0.05 when compared with sham group. ** p < 0.05 when compared with I/R group.

I/R caused significant (P < 0.05) elevation in kidney MDA level and significant (P < 0.05) reduction in kidney GSH level as compared with that of sham group. MK-886 significantly inhibited the elevated kidney MDA level and significantly increased the reduced kidney GSH level observed in I/R group. DITPA cause non-significant changes in kidney MDA and GSH levels as compared with that of I/R group (see Figure 3).

### Effects on Histology

**Figure 4:**
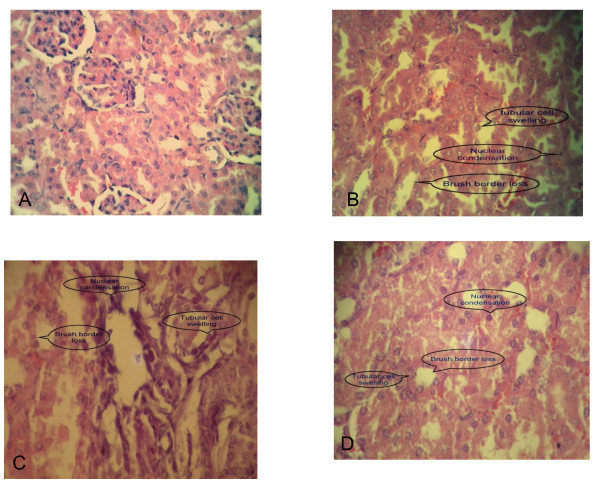
**Photomicrographs of kidney intersections; A show score 0; B show score 1; C show score 2; D show score 3. Sections stained with haematoxylin and Eosin (X 400)**.

The fallowing photomicrographs will show intersections of different severity scores obtained at the 48 hr after I/R (Figure 4).

**Figure 5:**
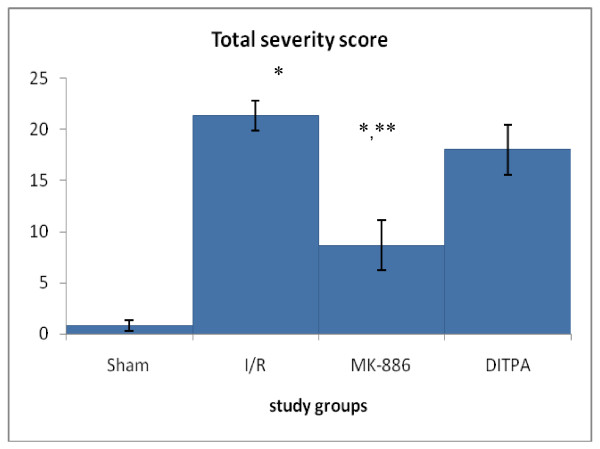
**Error bar chart shows the difference in mean ± SEM values of total severity scores in the four experimental groups at 48 hr after surgical operation (N = 6 in each group)**. * p < 0.05 when compared with sham group. ** p < 0.05 when compared with I/R group.

Histopathological changes resulted at the end of the study would be mentioned in table 1 and Figure 5. In sham group, most of slide sections of kidneys of mice in this group showed normal grading (normal architecture).

**Table 1:** The differences in histopathological grading of kidney changes among the four study groups at 48 hr after surgical operation.

Histopathological	Study groups							
	
changes grading	Sham		I/R		MK-886		DITPA	
	
	N	%	N	%	N	%	N	%
**Normal**	4	66.6	0	0	1	16.6	0	0

**Mild**	2	33.4	0	0	3	50	1	16.6

**Moderate**	0	0	2	66.6	2	33.4	3	50

**Severe**	0	0	4	33.4	0	0	2	33.4

**Total**	6	100	6	100	6	100	6	100

**Grade of the group**	**Normal**		**Severe**		**Mild**		**Moderate**	

In I/R group, slide sections of kidneys of four mice in this group showed severe grading. Mean of total severity scores of the sections of this group significantly higher than that of Sham group (P = 0.003). Treatment with MK-886 caused significant amelioration in kidney injury as seen by significant lowering in total severity scores of the sections of this group as compared with that of I/R group (p = 0.004). DITPA failed to get significant lowering

In total severity scores of the sections of this group as compared with that of I/R group (p = 0.292).

## Discussion

### Effects of Ischemia/Reperfusion

This study shows that IL-6 and TNF- α was significantly increased (P < 0.05) to more than that of sham group after I/R. Several studies show that I/R caused renal synthesis of the pro-inflammatory cytokines [9-12]. An explanation of this was that these cytokines are primarily produced by macrophage [46-48] and that I/R cause infiltration of macrophage to the kidney parenchyma [49]. **Takada *et al *(1997)**[9] were examined the initial (within 7 days) events of warm and in situ perfused cold ischemia of native kidneys in uninephrectomized rats. mRNA expression of the early adhesion molecule, E-selectin, peaked within 6 h; PMNs infiltrated in parallel. T cells and macrophages entered the injured kidney by 2-5 days; the associated upregulation of MHC class II antigen expression suggested increased immunogenicity of the organ. Th1 products (IL-2, TNF-α, IFNγ) and macrophage-associated products (IL-1, IL-6, TGF-β) remained highly expressed after 2 days [9]. For IL-6, **Kielar *et.al *2005**[50] found that in mouse model of ischemic renal injury, macrophages infiltrate the area of the vascular bundles of the outer medulla, these macrophages produce IL-6, and this IL-6 exacerbates ischemic murine acute renal failure [50]. Also **Sharyo *et al *(2009)**[42] found that serum IL-6 was elevated after 24 hr of reperfusion in 30 min ischemia and 48 hr reperfusion in mice [42].

I/R caused significant increase in oxidative status (increased generation of ROS). This finding agree with that in other studies: **Mejía-Vilet *et al *(2007)**[51] tested the effect of spironolactone on I/R rat model (20 minute kidney ischemia and 24 hours reperfusion) and they found that kidney MDA was significantly increased with significant decrease in kidney GSH in I/R group as compared with sham group [51]. **Seok *****et al *****(2007)**[52] show that I/R in mouse model (30 minute bilateral renal ischemia and 48 hours reperfusion) causing significant increase in kidney MDA and kidney GSH decrease [52].

In particular, at the onset of reperfusion, organs are exposed to an injurious burst of oxygen free radicals [53]. Natural antioxidant defenses may be overwhelmed and thus allow the oxygen radicals to exert their deleterious effects without control. Inflammatory cells involved in the damage produce more oxygen radicals, which increase the gravity of the initial injury [54]. This excess in ROS will lead to overcome antioxidant mechanisms and causing low level of tissue GSH and increased peroxidation of lipids and causing high tissue level of MDA (end product of lipid peroxidation)[55,56].

I/R caused significant impairment in kidney function as compared with that of sham group (due to low glomerular filtration rate [57]). I/R caused significant kidney injury due to IR-induced ROS production and nitric oxide in tubular epithelial cells beyond the scavenging capacity, and led to excessive ROS- and nitric oxide [19]-induced lipid peroxidation, DNA damage, and protein dysfunction leading to renal structural and functional impairment [18,19]. Also adhesion, activation, and transmigration of polymorphonuclear leukocytes (PMNs) into renal tissues and their oxidative burst resulted in excessive ROS production and worsening kidney damage (neutrophil-mediated tissue injury)[14,19]. Another mechanism is that TNF-α can cause renal tissue damage by direct cytotoxicity (induction of dysfunction and/or apoptosis)[58]. **Sharyo *et al *(2009)**[42] also showed significant increase in serum urea and creatinine levels after 30 min bilateral renal ischemia and 48 hr reperfusion [42]**. Seok *****et al *****(2007)**[52] showed that I/R in mouse model (30 minute bilateral renal ischemia and 48 hours reperfusion) revealed a severe loss of brush borders in the proximal tubular epithelial cells was observed, as well as luminal congestion, tubular atrophy, and tubular dilation in the outer medulla of the I/R mice [52].

### Effects of MK-886

The present study showed that the effect of use of MK-886 (6 mg/kg) before induction of ischemia and at the onset of reperfusion caused significant lowering (P < 0.05) in serum levels of the IL-6 and TNF-α. This could be explained by that MK-886 is a potent and selective inhibitor of FLAP [24]. So inhibit the biosynthesis of LTs including cysteinyl LTs and non cysteinyl LT (LTB_4_)[59]. **Poubelle *et al *(1991)**[60] clarify that LTB_4 _stimulated preferentially IL-6 production and that the observed LTB_4_-induced augmentation in thymocyte responses to monocyte supernatants is due to augmented IL-6 contents in the presence of baseline minimal IL-1 production [60]. **Rola-Pleszczynski *et al *(1992)**[61] reported that When human monocytes were cultured in the presence of graded nanomolar concentrations of LTB_4_, significant stimulation of production of bioactive and immunoreactive IL-6 was observed [61]. IL-6 regulates the expression of adhesion molecules and other cytokines in endothelial cells including IL-1β and TNF-α which in turn potently enhance the inflammatory response [62]. So inhibiting LTB_4 _synthesis (by MK-886) resulted in significant inhibition in IL-6 and TNF-α production. This conclusion about MK-886 effect on IL-6 and results obtained from the present study about effect of MK-886 in lowering IL-6 and TNF-α are consistent with that reported by **Cannetti *et al *(2003)**[63]that In an IL-18-dependent murine collagen-induced arthritis model, MK-886-treated mice displayed suppressed proinflammatory cytokine (IL-6 and TNF-α) production [63]. In that study, they mentioned that MK-886 act on IL-6 and TNF-α by the same mechanism supposed in this study although in different models.

MK-886-received animals had significant lower oxidative status (decreased generation of ROS) than that in I/R group animals. **Noiri *et al *(2000)**[21] demonstrated that LTB_4 _played a pivotal role in the recruitment of PMNs in kidneys subjected to I/R, and LTB_4 _receptor-antagonists abolished the PMN accumulation after I/R of the kidney [21]. Inhibiting LTB_4 _synthesis by MK-886 caused inhibition of activation of PMNs and their binding to endothelium by inhibiting the expression of cell-adhesion molecules and so block PMN infiltration into ischemic renal tissue, so inhibit release of ROS, proteases, elastase, myeloperoxidase (MPO), cytokines and various other mediators [19]. Also blocking the CysLTs synthesis, which are responsible of increased permeability, and thus, recruitment of neutrophils and macrophages, which are the producers of the pro-inflammatory mediators by MK-886 may contribute to inhibition of ROS release so less lipid peroxidation, less MDA level and less consumption of GSH and higher renal GSH level. By the same manner **Daglar *et al *(2009)**[27], in hepatic I/R in rat model, demonstrated that Inhibition of LTs action by montelukast or MK-886 has protective effects against I/R injury by reducing apoptosis and inhibition of ROS generation [27].

In this study, MK-886 causes significant preservation of renal function by inhibiting LTB_4 _and CysLTs synthesis, blocking PMN infiltration into ischemic renal tissue, so inhibiting the release of ROS and less TNF-α[13] so less production of NO (by inhibiting iNOS) so abolish lipid peroxidation, DNA damage, and protein dysfunction, leading to significant protection from renal structural and functional impairment [18,19]. **Şener *et al *(2006)**[28] showed that in rat model of I/R, Montelukast (10 mg kg^-1^, i.p.) administration at 15 min prior to ischemia and immediately before the reperfusion period caused significant lowering in creatinine and urea as compared with that of I/R group [28]. Also in the same study, montelukast significantly protected kidney parenchyma from severe damage observed in I/R group by inhibiting neutrophil infiltration, and regulating the generation of inflammatory mediators and ROS [28]. So it is not surprising for MK-886 to have protective effect on kidney parenchyma as it antagonizes the effect of not only the cystLTs but also LTB_4 _by inhibiting their biosynthesis.

It was noted that the renal injury and dysfunction observed in mice after I/R were not entirely abolished in mice received MK-886, the degree of inhibition of elevated serum urea and creatinine was not as complete as that of sham group. This is not surprising, given that many other pathophysiological mechanisms, which are independent of LTs and/or an enhanced inflammatory response, contributed to the observed injury during ischemia and/or reperfusion. These mechanisms may include (but are not limited to), modification of endogenous lipoxin generation [64] or the activation of the nuclear enzyme poly (ADP-ribose) polymerase [65].

### Effects of DITPA

To the best of our knowledge, this is the first study that used DITPA in model of renal I/R but TH (whether by acute T_3 _pretreatment or by long-term pretreatment with l-thyroxine)[31,32] and also DITPA were used in many models of I/R in several organs other than kidney and observed protective and/or improving action [37].

The possible mechanisms that underlie TH-induced cardioprotection are that Complex intracellular signalling underlies the cellular response to stress and the balance between pro-death and pro-survival pathways seems to determine cellular fate after an ischaemic insult. JNK kinases are thought as a pro-death signaling (in response to stressful stimuli). It was inhibited due to decreased phosphorylation due to reduced ATP availability (because of the reduced glycogen content which occurs during ischemia in hyperthyroid hearts)[34]. In the present study, DITPA, which has low metabolic activity, would not cause enough effect on ATP availability and so no inhibition of pro-death signals that is required to protect the kidney.

When myocardial ischemia was severe and induced irreversible damage, the improved post infarction function recovery by THs or DITPA may be mainly by their angiogenic action [36,66]. This action may have no role to play in reversible renal I/R model when inflammation play the major role in inducing injury and may explain why DITPA failed to exert protective effect in the reversible I/R model in this study.

Administration of DITPA after myocardial infarction in rabbits improved post-infarct recovery function [37]. Also In a rabbit post-infarction model, use of DITPA prevented abnormal sarcoplasmic reticulum Calcium transport (that mediate necrosis and permanent injury) in myocardial infarction [38]. So DITPA may not interfere with reversible I/R model in which inflammation rather than calcium dyshomeostasis have major role to play to cause tissue damage**s**.

In an organ other than heart, **Fernández *et al ***(2006)[67]showed that the use of T_3 _in I/R in liver (1 hr ischemia and 20 hr reperfusion) causing transient oxidative stress in the liver (occurred within a period of 36 hours of T_3 _treatment)[67]. This exerted significant protection against I/R injury, a novel preconditioning maneuver that is related to a gain of liver cell signaling functions represented by recovery of NF-κB and STAT3 DNA binding (NF-κB and STAT3 activation) and acute-phase response, which are lost during I/R. These responses may protect the liver against I/R-induced oxidative stress by re-establishing redox homeostasis [67]. Thyroid hormone pretreatment (10 μg/100 g body weight) 48 hr before I/R procedure significantly reduced parameters about oxidative stress compared with that of I/R group and so protected kidney from severe injury [68]. In the present study, DITPA administration was only 30 min prior to ischemia induction to see if there was any anti-inflammatory action for DITPA like that proved for MK-886 (before this study). This may be short time course to exert preconditioning effect on oxidative stress similar to that induced in hepatic and renal I/R injury in which T_3 _administration was 36 hr and 48 hr respectively prior to ischemia induction [67,68] and this may give suggestion for plane future in studying DITPA in I/R model.

## Conclusion

The results of the present study show that MK-886 significantly ameliorated kidney damage, resulted from I/R, by counteracting inflammatory and oxidative processes. DITPA, by this protocol of administration, failed to ameliorate kidney injury and the use of other administration way may give different effect on renal I/R injury.

## Competing interests

The authors declare that they have no competing interests.

## Authors' contributions

N R participated in the sequence alignment and drafted the manuscript, FG surgical aspect of experiment, AH participated in the design of the study and performed the statistical analysis, participated in its design and coordination. All authors read and approved the final manuscript.

## Pre-publication history

The pre-publication history for this paper can be accessed here:

http://www.biomedcentral.com/1471-2369/12/70/prepub
